# Measurement Method for Mold Slag Thickness in Continuous Casting Mold Using Millimeter-Wave Radar and Eddy Current Sensors

**DOI:** 10.3390/s26072141

**Published:** 2026-03-31

**Authors:** Yi An, Zhichun Wang, Junsheng Xiao

**Affiliations:** School of Automation and Electrical Engineering, Inner Mongolia University of Science & Technology, Baotou 014010, China; anyi19991107@163.com (Y.A.); xjsok@126.com (J.X.)

**Keywords:** millimeter-wave radar, eddy current sensor, mold slag thickness measurement, molten steel level detection, deep learning

## Abstract

To address the existing challenges in mold slag thickness measurement—such as the susceptibility of contact sensors to high-temperature degradation and the limitation of non-contact methods to detecting only the upper slag surface—this study proposes an integrated approach that fuses millimeter-wave radar and eddy current sensors for measuring mold slag thickness in a continuous casting mold. The method innovatively combines two sensing principles: the millimeter-wave radar employs an improved FFT-CZT^2^ high-precision ranging algorithm to perform high-resolution scanning of the solid slag upper surface, reconstructing its topography (error: ±1 mm), while Mel-frequency cepstral coefficients (MFCC) are applied to extract features from the radar intermediate-frequency signals, combined with an enhanced PSO-BP neural network algorithm to predict the thickness of the solid slag layer (error: ±5 mm). Concurrently, an eddy current sensor monitors the liquid slag–molten steel interface position (error: ±1 mm). Through dual-sensor data fusion, the upper surface topography data and solid slag thickness obtained from the radar are spatially registered in three dimensions with the molten steel level information derived from the eddy current sensor. This integration ultimately enables the non-contact synchronous measurement of three key parameters within the mold: solid slag layer thickness, liquid slag layer thickness inversion, and molten steel level. Furthermore, by reconstructing the upper slag surface morphology, the method successfully resolves practical issues such as uneven material distribution, local material deficiency, or excessive feeding. Preliminary experimental verification confirms that the proposed method maintains stable performance even under high-temperature and complex environmental conditions. It thus provides a real-time, accurate, and full-cross-section monitoring solution for mold slag in continuous casting, offering significant practical value for the development of smart steel plants.

## 1. Introduction

The mold is the core equipment in continuous casting, responsible for the initial solidification of molten steel and the formation of the solidifying shell. Mold slag serves multiple functions, including thermal insulation, inclusion absorption, oxidation prevention, and lubrication. After molten steel is poured into the mold, it contacts the water-cooled copper wall and forms a solidified layer. Under static pressure, the solidifying shell tightly adheres to the copper wall, continuously transferring heat and increasing in thickness. Mold oscillation causes the shell to separate from the copper wall, forming an air gap, which reduces heat transfer. The molten slag fills this air gap, enhancing heat transfer while providing lubrication, thereby reducing withdrawal resistance and allowing the strand to smoothly enter the secondary cooling zone. During continuous casting, slag must be continuously replenished to maintain a dynamic thickness balance. The thickness of the mold slag directly affects both heat transfer and lubrication efficiency, making it critical for strand quality and continuous casting stability.

Currently, the detection methods for mold slag thickness are primarily categorized into contact and non-contact types. The traditional contact method typically employs a measuring rod made of high-temperature-resistant material. The rod is inserted into the slag layer for a brief period and then withdrawn, allowing for a rough estimation of layer thickness by observing the color changes on the rod. While simple to operate, this method is susceptible to human factors and material properties, leading to significant measurement inaccuracies. Furthermore, researchers [1,2] have proposed a machine-vision-based method for mold slag layer thickness detection and validated its reliability. This technique also uses a high-temperature-resistant measuring rod inserted into the slag. After withdrawal, machine vision is employed to extract the temperature distribution characteristics from the rod. The thickness is then determined by correlating these characteristics with the known temperature profiles of different slag layers inside the mold. The advantage of this method lies in its ability to effectively mitigate interference from environmental factors. However, its application remains limited by insufficient real-time performance and the high cost of the required high-temperature-resistant materials. The cited work indicates that the error in detecting the molten steel meniscus position is less than 5 mm, but it does not provide an error assessment for the solid slag layer thickness. Literature [3] developed an online measurement device for molten slag layer thickness. Based on the principle of electrical conductivity variation, the device measures the slag at the mold’s edge, quarter point, and center. The reported molten slag layer thicknesses at these positions were 4.7 mm, 11.2 mm, and 18.4 mm, respectively, with a total slag thickness (solid and molten layers) ranging from 45 to 50 mm. While this study is of significant importance, its experiments were conducted in an actual industrial environment without an in-depth analysis of measurement errors. Moreover, the electrodes used are prone to erosion in the molten slag and steel environment, making it difficult to guarantee long-term measurement stability and accuracy.

Non-contact measurement methods provide an effective approach for mold slag thickness detection, eliminating the reliance on high-temperature-resistant materials. Literature [4] developed a slag feeding robot equipped with real-time slag thickness detection and feedback control functionality. The system incorporates a laser rangefinder sensor designed to measure the slag thickness in real-time, with a stated measurement accuracy of 1 mm, although no additional disclosure regarding measurement error was provided. However, this system is limited to measuring the distance to the upper surface of the solid slag layer, from which the solid slag thickness is derived. The overall control error for the total slag thickness was reported to be manageable. In practical application, it can maintain the mold slag thickness within the specified process requirement range of 35–50 mm, with no reported deviations exceeding this interval. Literature [5] proposed a method for measuring solid slag layer thickness using a vector network analyzer for microwave time-domain measurements. This method was successfully applied to measure powder thickness on an aluminum plate with an error of 0.56 mm. However, the paper noted that the method failed to detect the reflected wave from the bottom surface of the liquid slag layer. Literature [6] presented research on real-time molten steel level and mold slag thickness detection based on radar technology. The study experimentally verified the feasibility of using ultra-wideband (UWB) radar for slag layer thickness measurement but did not thoroughly discuss the accuracy of the experimental results or the measurement errors. Literature [7] introduced a mold slag thickness measurement method based on ultra-wideband (UWB) radar technology. The system employed a vector network analyzer (VNA) and a broadband horn antenna, operating in the frequency range of 10 GHz to 40 GHz in a frequency-stepped continuous wave (FSCW) mode. Data acquisition and processing could be completed within 500 Ms. Nevertheless, the study did not discuss the measurement errors or the impact of low-frequency signal attenuation on accuracy.

Millimeter-wave radar finds increasingly widespread applications in daily life and medical fields, encompassing areas such as human gesture recognition [8,9,10], personnel identification [11], vital sign detection [12,13], static material identification [14], and object detection [15]. In the field of non-destructive testing of materials, millimeter-wave radar technology demonstrates broad application potential due to its superior penetration capability [16,17]. D. Meier, A. Och et al. [18,19,20] utilized the penetration capability of millimeter-wave radar and its high sensitivity to phase variation to achieve defect detection and tomographic imaging in composite materials. K. Dausien et al. [21] integrated millimeter-wave radar with material characterization algorithms to realize accurate ranging and material imaging, while also pointing out challenges posed by near-field wave propagation effects and environmental factors on measurement accuracy. X. Zhang et al. [22] independently developed an all-solid-state electronic three-dimensional millimeter-wave imaging radar, which was applied to the non-destructive testing of polymer materials. Their work verified the effectiveness of radar in detecting both surface and internal defects.

In the aspect of molten steel level detection within the mold, Katankin et al. [23] investigated the eddy current method for thin-slab continuous casting. Compared to radioactive techniques, the eddy current method offers advantages such as insensitivity to slag, rapid detection, and environmental friendliness. Through ANSYS (2022R2) simulations, the study validated that employing a 10 mm copper shield (with an excitation current of 1 A and a frequency of 2500 Hz) can concentrate the magnetic field and suppress interference. A comparison of three orthogonal excitation windings revealed that EW-1 achieved optimal sensitivity on the mold surface, while EW-2 exhibited a stronger signal at the mold bottom. Analysis indicated that temperature variations in the probe affect the signal via electrical conductivity, and increasing the gap between the probe and the copper plate can mitigate temperature-related interference. This research provides a theoretical and simulation foundation for the engineering application of eddy current detection probes. I. V. Terekhin et al. [24] proposed a direct method using an array eddy current sensor for molten steel level detection in continuous casting molds. This method directly locates the metal meniscus by analyzing the signal distribution characteristics received by the sensor coils, enabling self-calibration and rapid measurement. It overcomes the limitations of traditional calibration methods, which struggle to adapt to variable operating conditions, and offers a novel approach in this field. Experiments demonstrated its effectiveness in suppressing interference from slag and temperature rises in the copper wall, though dynamic factors such as electromagnetic stirring require further investigation.

Thus, we propose an integrated method for measuring mold slag thickness within a continuous casting mold by fusing 77 GHz millimeter-wave radar and eddy current sensors. Our approach introduces a millimeter-wave radar sensor to reconstruct the upper surface topography and predict the thickness of the solid slag layer, while simultaneously employing an eddy current sensor to achieve precise measurement of the molten steel level. By performing three-dimensional spatial registration of the dual-sensor data, comprehensive cross-sectional monitoring within the mold is enabled. This provides a feasible solution to address the limitations of traditional methods, which are significantly affected by high-temperature molten steel and are often restricted to measuring only the height of the upper surface of the solid slag layer.

The contributions of this work are as follows:

(1) The improved FFT-CZT^2^ high-precision ranging algorithm is applied to reconstruct the topography of the upper surface of the solid slag layer, effectively addressing issues such as uneven material distribution, local material deficiency, and excessive feeding.

(2) In an innovative application, Mel-frequency cepstral coefficients (MFCC) are utilized across domains to extract features from radar intermediate-frequency signals. This approach, combined with an improved PSO-BP neural network (PSO-BPNet) algorithm, enables high-precision prediction of the solid slag layer thickness.

(3) The molten steel level is precisely measured using an eddy current sensor, enabling the inversion of the key parameter—liquid slag layer thickness—within the mold.

The rest of the paper is organized as follows: System Simulation Experiments and Related Algorithm Enhancements are introduced in Section 2. The experimental details and results are provided in Section 3. Finally, the conclusion and future work are given in Section 4.

## 2. Methodology

### 2.1. Overall System Framework

**Figure 1:**
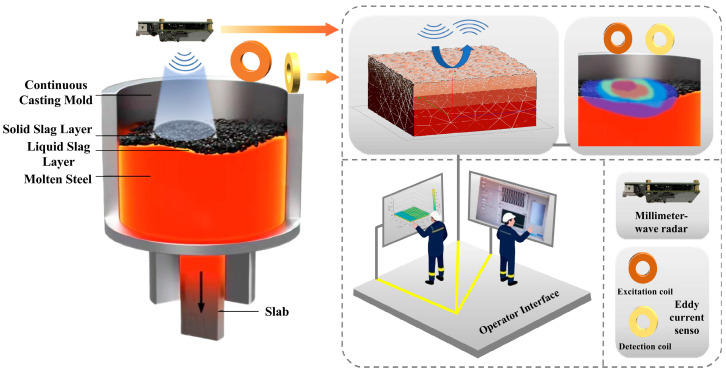
Overall System Framework.

This paper proposes an overall system framework for measuring mold slag thickness in a continuous casting mold based on the fusion of millimeter-wave radar and eddy current sensors, as illustrated in Figure 1. The system employs a dual-sensor fusion framework, encompassing core modules such as topography reconstruction of the solid slag layer’s upper surface, prediction of solid slag layer thickness, detection of the molten steel level, and inversion of the liquid slag layer thickness.

### 2.2. System Simulation Experiments

#### 2.2.1. Millimeter-Wave Radar Simulation for Mold Slag Detection

This section employs simulation experiments to evaluate the feasibility of using millimeter-wave radar for detecting the thickness of the solid slag layer.

Millimeter-wave radar operates by transmitting and receiving electromagnetic waves for detection. When these waves propagate to a target, phenomena such as transmission, attenuation, and refraction occur, generating echo signals that contain information about the target’s radar cross-section (RCS). The RCS is a key parameter characterizing the target’s ability to reflect electromagnetic waves, with its value jointly influenced by the target’s geometric structure, surface characteristics, and material properties.

Since the solid slag layer inside the mold exhibits weak conductivity, millimeter-wave signals can effectively penetrate it. However, the intensity and characteristics of the echo signals vary with the thickness of the mold slag. Essentially, the echo signals reflect the electromagnetic reflection properties of the measured material, yet their strength is influenced by multiple factors such as electrical conductivity, thickness, dielectric constant, and surface roughness. Based on this principle, radar is capable of not only detecting the distance and motion state of a target but also further extracting information about the material’s properties, thickness, and surface topography by analyzing the characteristics of the echo signals. This enables effective thickness detection.

**Figure 2:**
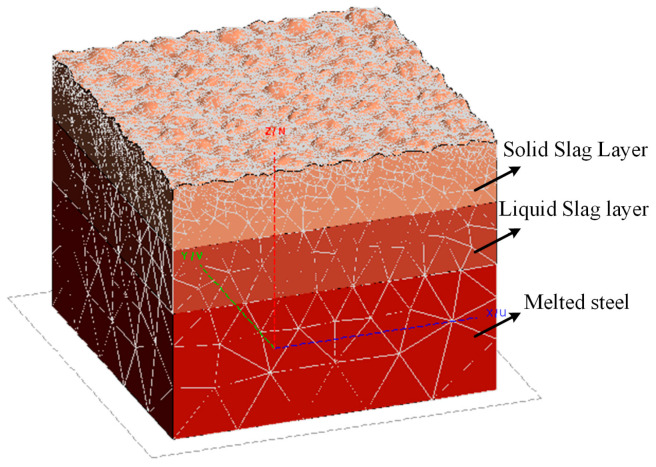
Three-Dimensional Geometric Model Construction.

A three-dimensional geometric model incorporating the solid slag layer, liquid slag layer, and molten steel was established using FEKO (2022.1) simulation software to calculate its radar cross section (RCS) and transmission coefficients. The 3D model is shown in Figure 2. In the simulation analysis, the thickness of the solid slag layer was set to vary from 1 to 4 cm, a range based on typical observed values of the solidified slag layer thickness under actual continuous casting conditions. The thickness of the liquid slag layer was fixed at 1 cm, corresponding to the liquid slag layer thickness in practical operation. The thickness of the molten steel layer was set to 2 cm, primarily due to the significant attenuation of radar electromagnetic waves when penetrating molten steel, which prevents the detection of deeper regions in practice. This simplification also helps reduce the computational load.

The upper surface of the solid slag layer is a randomly rough surface, generated based on COMSOL (COMSOL 6.1) software. It can be regarded as the superposition of multiple fundamental waves with different spatial frequencies and phase angles. The expression for these fundamental waves is as follows:

(1) $$f(x,y)=\sum_{mn}\alpha (m,n)\cos(2\pi (mx+ny)+\varphi (m,n))$$

where α(m,n) denotes the amplitude, φ(m,n) denotes the phase angle. The amplitude is generated following a Gaussian distribution, while the phase angle is randomly generated within the range [−π/2,π/2].

The procedure for generating the rough surface is as follows: define key parameters in COMSOL, such as spatial frequency and spectral index; generate random amplitudes and phase angles based on a Gaussian distribution; and construct a parameterized random rough surface using Equation (2).

(2) $$Z=0.01\sum_{m=-N}^{N}\sum_{n=-N}^{N}if(m!=0)||(n!=0),({\left(m^{2}+n^{2}\right)}^{-\frac{b}{2}}g_{1}(m,n)\cos(2\pi (mx+ny)+u_{1}(m,n)),0)$$

In the COMSOL software, Equation (2) is used to generate a parameterized random rough surface with specific statistical properties. This method is implemented through numerical approaches and can be directly constructed and executed within the software environment, where m,n is the wavenumber index, with a value range of [−N,N]; b is the spectral index; $g_{1}$ represents the amplitude function; $u_{1}$ represents the phase angle function. Subsequently, using the “Cut Domain” operation within the geometry module, the initial geometry is partitioned to generate a three-dimensional geometric model featuring a rough upper surface.

**Figure 3:**
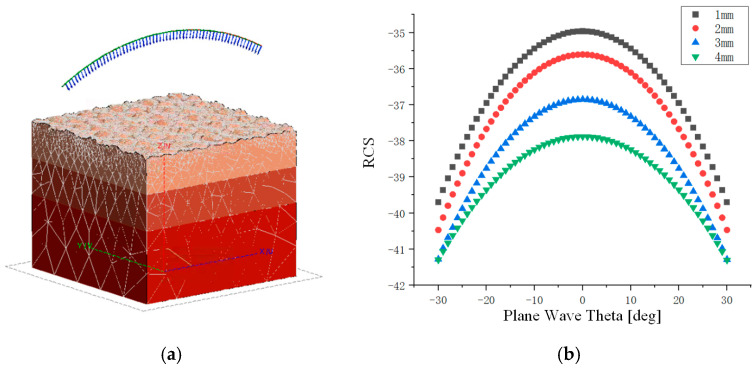
Influence of Different Solid Slag Layer Thicknesses on RCS Values. (**a**) Simulation Model of Millimeter-Wave Radar Illuminating Mold Slag. (**b**) Effect of Thickness on RCS Value Curves.

In the geometric model, the dielectric constants of different media are assigned, and the thickness of the solid slag layer is sequentially set from 1 to 4 cm. After completing the mesh generation, the model is imported into FEKO software, where a monostatic excitation signal, incident angle, and solver parameters are configured to perform radar cross-section (RCS) simulation calculations. The simulation results indicate that as the thickness of the solid slag layer increases, the RCS value gradually decreases. The simulation results are shown in Figure 3.

**Figure 4:**
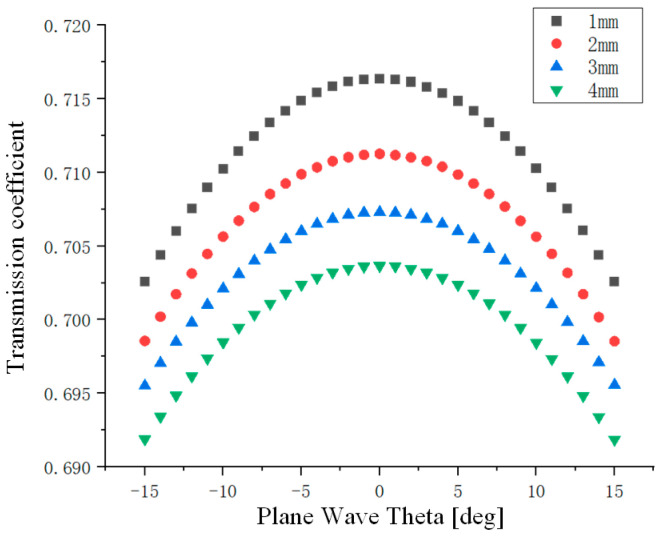
Influence of Different Solid Slag Layer Thicknesses on Transmission Coefficient.

Transmission characteristics simulation was conducted on the multilayer medium structure comprising the solid slag layer, liquid slag layer, and molten steel. By configuring the corresponding parameters for each medium layer, selecting periodic boundary conditions and specifying two-dimensional extension directions, and defining a monostatic excitation signal, incident angle, and solver, the transmission characteristic analysis was completed. The simulation results show that as the thickness of the surface solid slag layer increases, the transmission efficiency of the electromagnetic wave signals gradually decreases. The simulation results are presented in Figure 4.

#### 2.2.2. Simulation of Molten Steel Level Detection Using Eddy Current Sensors

This paper presents the optimization and design of an eddy current sensor through simulation experiments. A stepwise optimization strategy is employed to determine the key parameters. Due to the strong coupling among parameters such as excitation frequency, probe geometry, and coil position, a simultaneous global optimization approach would result in an excessively large solution space and prohibitively high computational costs. Therefore, the optimal excitation parameter combination is first determined through basic simulations, followed by the stepwise optimization of geometric parameters such as coil radius and number of turns. It should be noted that the stepwise optimization approach can be considered a limitation of this study, which will be addressed in future work.

Due to the structural complexity of the model, a three-dimensional spatial simulation model was established in COMSOL simulation software to enhance the accuracy of the simulation results. To emulate the actual working environment, an infinite element domain was configured in the model to simulate the unbounded diffusion behavior of the magnetic field in space.

**Figure 5:**
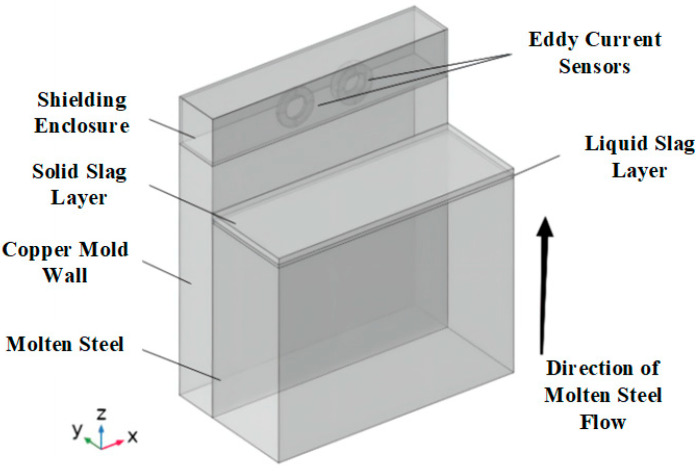
Structure of the Eddy Current Sensor Simulation Model.

This three-dimensional simulation model primarily consists of eight components: the excitation coil, used to generate the primary magnetic field; the detection coil, used to receive the induced voltage signal from eddy currents; the solid slag layer, liquid slag layer, and molten steel layer, collectively simulating the actual working conditions of the continuous casting process; the copper wall, used to simulate the structure of the mold wall; the shielding enclosure, employed to suppress external interference; and the infinite air domain, utilized to simulate the free divergence behavior of the magnetic field in space. The model structure is shown in Figure 5.

In practical applications, a mold specification of approximately 700 mm is commonly employed. The initial simulation parameters for the mold and its internal media used in this study are summarized in Table 1.

1. Excitation Selection for the Sensor

A sinusoidal wave was selected as the sensing signal, and its amplitude and frequency parameters were optimized through simulation. The induced voltage in the detection coil was analyzed under excitation amplitudes of 5 V, 8 V, and 10 V. The peak induced voltage showed a positive correlation with the excitation amplitude, with a maximum difference of approximately 130 mV. The signal variation under 5 V excitation was too small to be reliably detected or resolved experimentally, whereas the 10 V excitation produced a higher induced voltage with the most pronounced signal variation, meeting the detection requirements. Considering the effective output range of the FY6300 signal generator, 10 V was ultimately selected as the experimental excitation amplitude.

To investigate the influence of excitation frequency on the detection signal, multiple sets of simulations were conducted under the condition of fixed molten steel position and other parameters, with a starting frequency of 100 Hz, a final frequency of 5000 Hz, and a step size of 200 Hz. By analyzing the response characteristics of the induced voltage at different frequencies, the optimal excitation frequency was selected.

**Figure 6:**
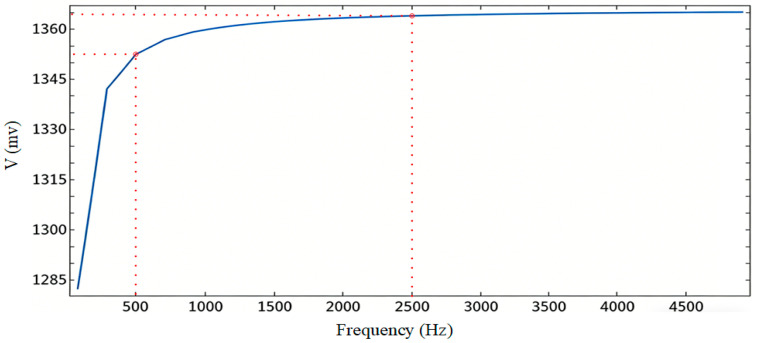
Induced Voltage versus Excitation Frequency. The figure presented is a direct output from the COMSOL simulation software.

As shown in Figure 6, the amplitude of the induced voltage increases with the excitation frequency, but the growth trend varies across different frequency ranges: it rises relatively rapidly below 500 Hz, slows down within the 500–2500 Hz interval, and stabilizes beyond 2500 Hz. This indicates that the voltage amplitude approaches an upper frequency limit. Building on this, the controlled variable method was employed (with model parameters fixed and only the excitation frequency varied) to measure the change in induced voltage in the detection coil as the molten steel distance varied within the range of 50–160 mm, as summarized in Table 2.

According to Table 2, the induced voltage variation decreases as the excitation frequency increases. When the frequency is below 1000 Hz, the variation is larger, indicating higher detection sensitivity, and the differences in variation are small. Considering both sensitivity and mainstream level meter design, 800 Hz is ultimately selected as the excitation frequency.

2. Coil Parameter Selection

The geometric parameters of the coil (e.g., outer diameter, inner diameter) directly affect the detection sensitivity and measurement range of the sensor. Using COMSOL finite element simulation, the impact of coil geometric parameters on sensor performance is systematically analyzed.

A. Selection of Coil Outer Diameter

**Figure 7:**
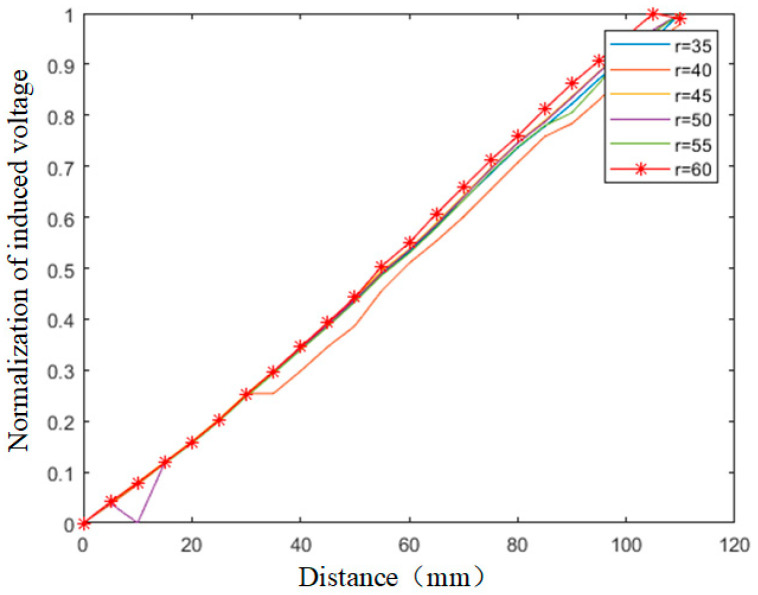
Influence of Coil Radius on Induced Voltage.

Increasing the coil size helps enhance penetration depth but reduces sensitivity. Due to the relatively large dimensions of the mold and the significant distance to the molten steel (50–160 mm), an excessively small coil outer diameter would result in excessively weak eddy current signals, hindering detection. Therefore, simulations analyzed the variation in coil radius within the range of 35 mm to 60 mm, and the normalized induced voltages are shown in Figure 7.

**Figure 8:**
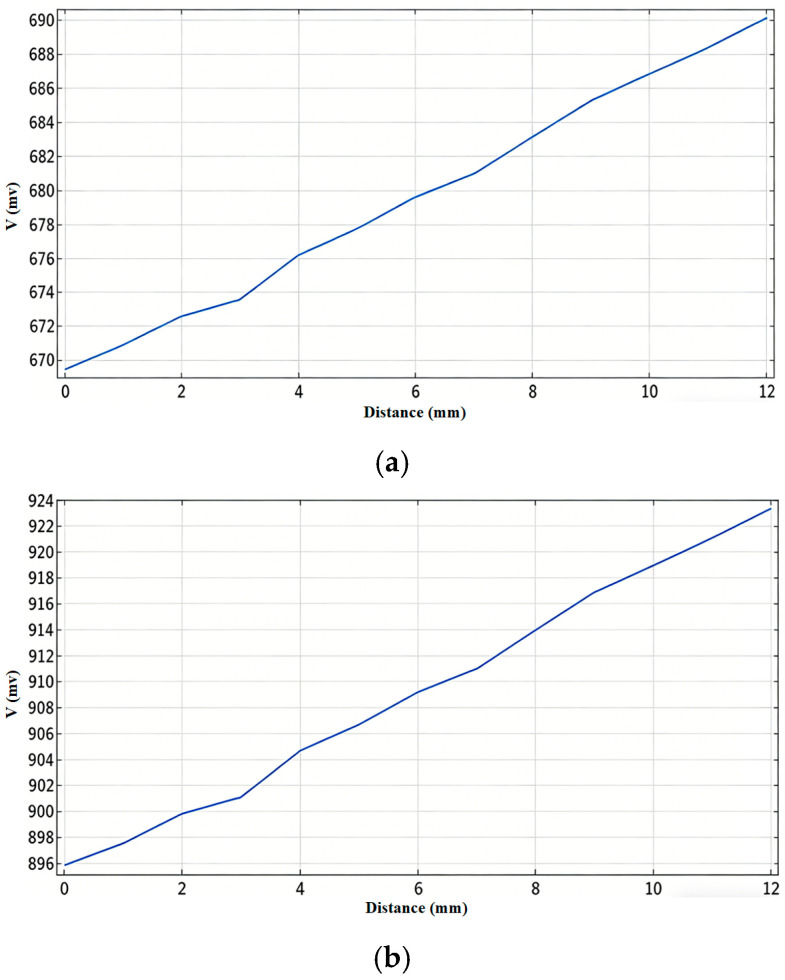
Effect of Coil Radius on Induced Voltage. (**a**) Induced voltage curve for a coil radius of 55 mm. (**b**) Induced voltage curve for a coil radius of 60 mm. The figure presented is a direct output from the COMSOL simulation software.

Based on the analysis of the above data, when the molten steel distance is within 60 mm, variations in coil radius have a minor impact on the detection results. As the distance increases, the slope of the normalized induced voltage curve gradually rises, indicating improved detection sensitivity. To guarantee overall detection accuracy, separate simulations are performed using coil radii of 55 mm and 60 mm as preferred values. The induced voltage curves and the magnetic field distribution at a molten steel distance of 160 mm are shown in Figure 8.

Based on the analysis of the data in Figure 8, when the coil radius is 55 mm, the overall change in induced voltage corresponding to a 120 mm variation in the molten steel level is approximately 20 mV; for a radius of 60 mm, the voltage change is about 30 mV. At a liquid level of 160 mm, the magnetic field intensities generated by the two coils are 45 × 10^−7^ T and 5 × 10^−6^ T, respectively, with values being relatively close. Considering both detection sensitivity and linear range, a radius of 60 mm is ultimately selected for both the excitation and detection coils.

B. Selection of Coil Inner Diameter

**Figure 9:**
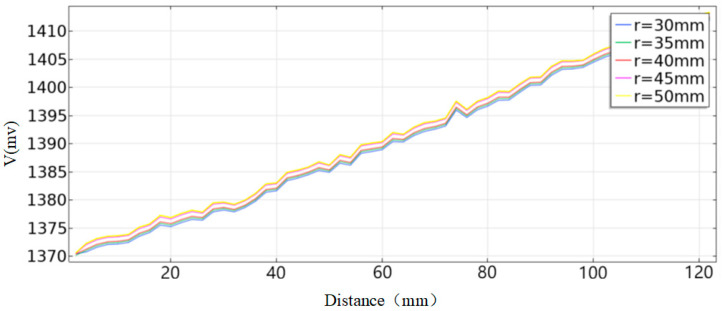
Effect of Coil Inner Diameter Variation on Induced Voltage.

Keeping other simulation parameters constant, the inner radius of both the excitation and detection coils was increased from 30 mm to 50 mm. The resulting induced voltage curves for different inner radii are shown in Figure 9.

The results indicate that as the inner diameter varies within the range of 30 mm to 50 mm, the total variation in induced voltage remains largely consistent. Under the premise of a constant number of coil turns, an increase in inner diameter leads to a reduction in coil thickness. A comparison reveals that the induced voltage curves for inner diameters of 45 mm and 50 mm are highly consistent. Taking into account structural parameters such as outer diameter, wire diameter, and number of turns, an inner diameter of 45 mm is ultimately selected for both the excitation and detection coils.

3. Placement Configuration of Sensor Coils

**Figure 10:**
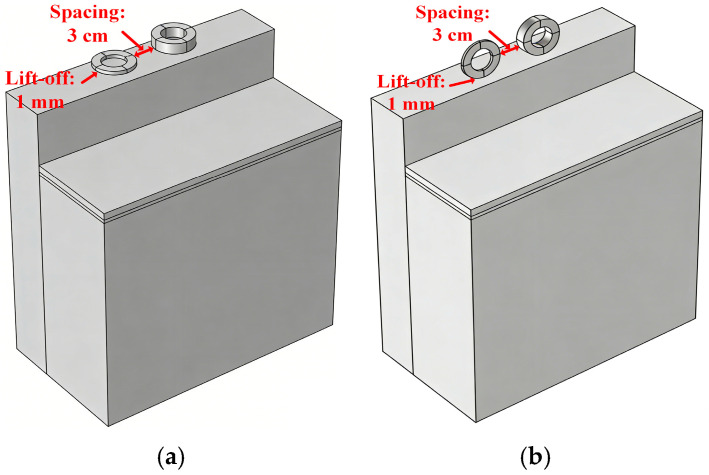
Coil Placement Configuration. (**a**) Coils parallel to the copper wall. (**b**) Coils perpendicular to the copper wall.

Two placement schemes are proposed for the coils: (a) coils parallel to the copper wall; (b) coils perpendicular to the copper wall, as illustrated in Figure 10. The coil parameters are listed in Table 1.

**Figure 11:**
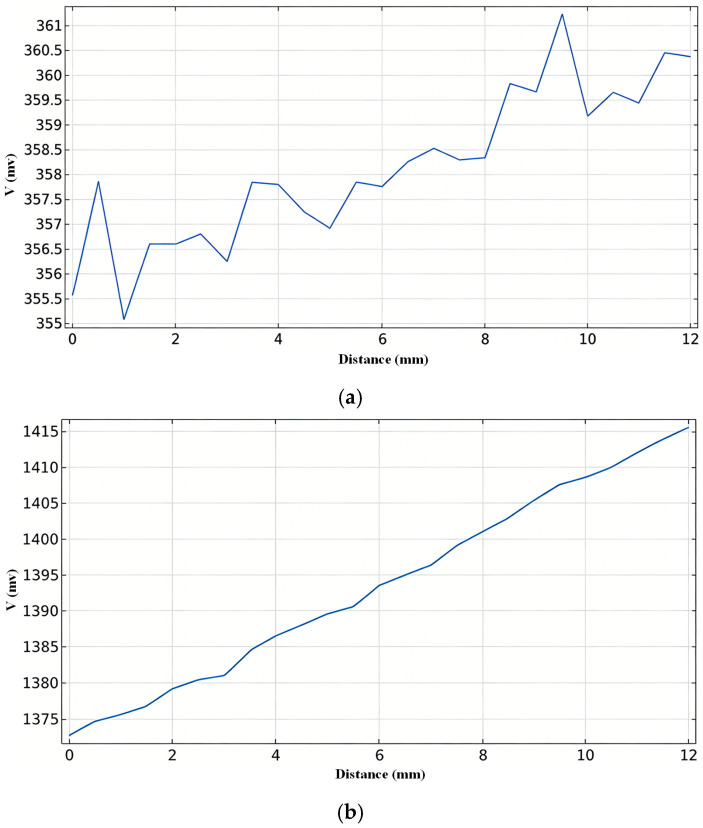
Effect of Coil Placement Configuration on Induced Voltage. (**a**) Coils parallel to the copper wall. (**b**) Coils perpendicular to the copper wall.

Keeping the coil parameters unchanged, simulations were conducted for different positions of the sensor coils, yielding various induced voltage curves. The results are shown in Figure 11.

Figure 11 presents a comparison of the experimental results for two different coil placement schemes, visually demonstrating the critical role of the optimized design in enhancing detection performance. Based on the induced voltage data in Figure 11, under the same variation in molten steel level, the voltage change for scheme (a) is approximately 3 mV, while for scheme (b) it reaches about 55 mV. The comparison indicates that although the voltage in scheme (a) shows an overall increasing trend, it exhibits irregular changes during small-scale level fluctuations, resulting in detection errors far exceeding the design target. In contrast, the induced voltage in scheme (b) demonstrates a good linear relationship across the entire measurement range; even minor level changes produce a noticeable response, which is more conducive to achieving precise detection. Therefore, the detection performance of scheme (b) is significantly superior.

### 2.3. Related Algorithms and Their Improvements

To achieve non-contact, full-section, and simultaneous measurement of the slag layer (solid and liquid) and the molten steel level inside the mold, this section establishes a comprehensive core algorithm framework. Section 2.3.1 utilizes the characteristics of millimeter-wave radar echo signals and adopts the FFT-CZT^2^ high-precision ranging algorithm to perform high-resolution scanning of the solid slag surface, accurately reconstructing its burden topography. This method provides a spatial reference for subsequent computations and supports the diagnosis of process issues such as uneven material distribution. To realize the inversion of solid slag thickness, Section 2.3.2 applies Mel-frequency cepstral coefficients to process the radar intermediate-frequency signals for extracting implicit features. Section 2.3.3 employs a PSO-BPNet model for prediction, with this optimization strategy significantly enhancing the convergence speed and generalization capability of the neural network. Concurrently, Section 2.3.4 independently implements molten steel meniscus monitoring based on the eddy-current detection principle, thereby supplying a stable molten steel level reference for the slag thickness measurement system.

#### 2.3.1. FFT-CZT^2^ High-Precision Ranging Algorithm

The FFT-CZT^2^ high-precision ranging algorithm is a cascaded spectral analysis method improved upon the traditional FFT [25] and CZT [26] algorithms, designed to achieve super-resolution frequency estimation. The algorithm significantly enhances measurement accuracy through a three-stage progressive processing architecture:

Step 1: Coarse Frequency Estimation Based on FFT

An N-point FFT operation is performed on the N-point ADC sampled sequence, with a frequency resolution of $\Delta f=f_{s}/N$, where $f_{s}$ is the sampling rate. The frequency ($f_{FFT}$) corresponding to the peak amplitude in the FFT spectrum serves as the coarse frequency estimate. Under ideal conditions, if the target frequency $f_{FFT}$ is an integer multiple of Δf (i.e., $f_{FFT}=k\Delta f$), the target distance can be directly obtained from the target frequency. Otherwise, the maximum error in frequency estimation is ±Δf/2, and the theoretical expression for ideal frequency estimation is given by Equation (3):

(3) $$f_{ideal}=f_{FFT}\pm \frac{f_{s}}{2N}$$

Step 2: Fine Frequency Estimation Based on CZT

To enhance frequency estimation accuracy, the CZT is employed for spectral analysis within a narrowband range [$f_{lower\_czt}$, $f_{upper\_czt}$], where the narrowband range formula is given by Equation (4):

(4) $$f_{lower\_czt}=f_{FFT}-\frac{f_{s}}{N},\;f_{upper\_czt}=f_{FFT}+\frac{f_{s}}{N}$$

The transform kernel parameters A and W for CZT are defined by Equation (5):

(5) $$A=\exp\left(\frac{j2\pi f_{lower\_czt}}{f_{s}}\right),\;W=\exp\left(\frac{-j2\pi (f_{upper\_czt}-f_{lower\_czt})}{Mf_{s}}\right)$$

where M is the number of CZT points. The frequency ($f_{CZT}$) corresponding to the peak in the CZT spectrum is more accurate than $f_{FFT}$, and the theoretical expression for ideal frequency estimation is given by Equation (6):

(6) $$f_{ideal}=f_{czt}\pm \frac{f_{s}}{2NM}$$

Step 3: Iterative CZT Optimization and Precision Limit

Theoretically, the theoretical expression for ideal frequency estimation after k iterations is given by Equation (7):

(7) $$f_{ideal}=f_{czt^{\left(k\right)}}\pm \frac{f_{s}}{2NM^{k}}$$

In practical systems, factors such as multipath interference, noise, and spectral leakage introduce additional errors, limiting the achievable maximum accuracy. Experimental results indicate that two iterations of CZT are sufficient to approach this limit, with further iterations providing no significant improvement in precision.

#### 2.3.2. Mel-Frequency Cepstral Coefficients (MFCC)

The Mel-Frequency Cepstral Coefficients (MFCC) [27], a classic feature extraction method in speech signal processing, is cross-applied to millimeter-wave radar signal analysis. The theoretical basis for this lies in the fact that both speech signals and millimeter-wave radar signals are frequency-modulated (FM) signals, sharing essential similarities in their time-frequency characteristics.

The core advantage of MFCC lies in its bio-inspired design concept: it simulates the human ear’s sensitivity to frequency through mel-scale filtering, thereby extracting more representative spectral features that are easier to distinguish and process in subsequent prediction tasks. Additionally, MFCC features focus on describing the qualitative characteristics of the signal, offering strong robustness. Compared to directly using time-domain or frequency-domain signals, MFCC features can better characterize the frequency reflection properties of the target object. The computational steps are as follows:

Step 1: Pre-emphasis: The backscattered signal is passed through a filter to enhance high-frequency components, boosting the energy of high-frequency signals and mitigating the effects of high-frequency attenuation during signal transmission.

(8) X(n)=X[n]−αX[n−1] 0.9≤α≤1.0

where X[n] is the pre-emphasized signal; α is the pre-emphasis factor.

Step 2: Windowing: The pre-emphasized signal is multiplied by a window function to maintain signal continuity and reduce spectral leakage.

(9) Y(n)=X(n)×W(n)

(10) $$W(n)=0.54-0.46\cos(\frac{2\pi n}{N-1})\;0\leq n\leq N-1$$

where the window function is selected as the Hanning window function (W(n)); N is the number of samples per frame; 2πn is the angular frequency.

Step 3: Fast Fourier Transform (FFT): The N samples in each frame are transformed from the time domain to the frequency domain, yielding the spectral energy distribution, which facilitates better observation and analysis of the signal characteristics.

(11) $$X(k)=\sum_{n=0}^{N-1}x(n)w_{e}(n)e^{\frac{-j2\pi nk}{N}}$$

where X(k) represents the spectrum; x(n) represents the time-domain sampled data; $w_{e}(n)$ represents the window function; N represents the Fourier length.

**Figure 12:**
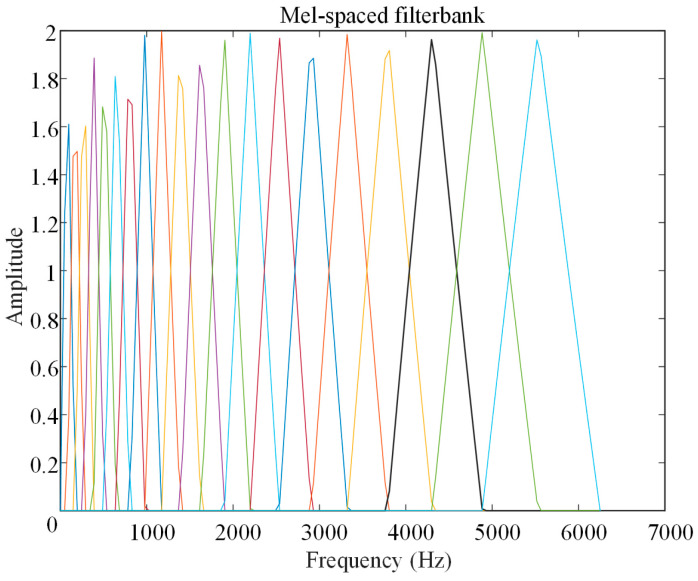
Mel Filter Bank.

Step 4: Mel Filter Bank: Figure 12 illustrates a mel filter bank composed of 100 non-uniform triangular bandpass filters. The spectrum is multiplied by these filters, and the energy for each frame is summed. Mel filter banks are feature transformation tools used for processing millimeter-wave radar spectra. Their function is to map the linear spectrum to the mel frequency domain to extract band energy features that are more effective for prediction tasks. The specific parameters are shown in Table 3.

**Figure 13:**
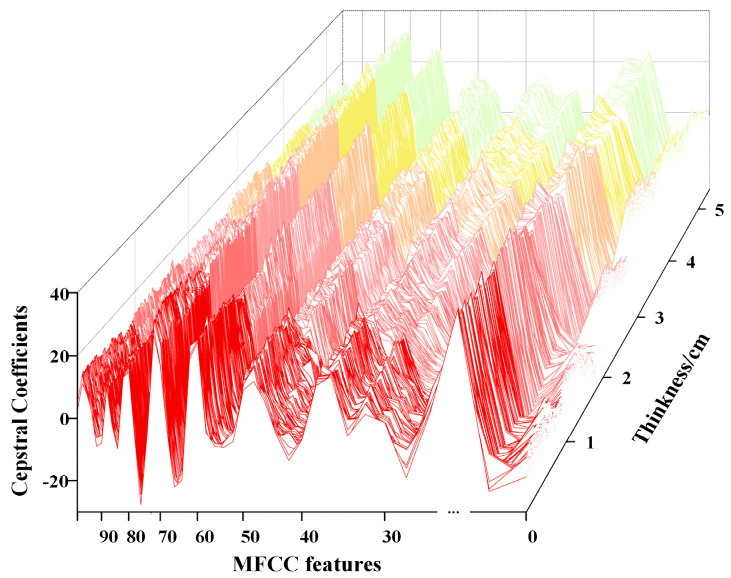
MFCC Visualization Results.

Step 5: Discrete Cosine Transform (DCT): The logarithmic mel spectrum is transformed into the time domain using the discrete cosine transform to obtain the Mel-Frequency Cepstral Coefficients (MFCC). Figure 13 presents the visualization results of MFCC for a sample, with each sample extracting 100-dimensional coefficients.

MFCC is a core feature of the intermediate-frequency signal in millimeter-wave radar. It converts the logarithmic Mel spectrum into the time domain through Discrete Cosine Transform (DCT), retaining the key perceptual information related to solid slag layer thickness while achieving feature decorrelation. This visualization clearly reveals the numerical distribution of 100-dimensional features across samples of different solid slag layer thicknesses, as well as the characteristic patterns of these samples. This provides interpretable feature evidence for subsequent thickness prediction.

#### 2.3.3. PSO-BPNet

The PSO-BPNet architecture innovatively integrates the Particle Swarm Optimization (PSO) algorithm with the Backpropagation (BP) [28] neural network, constructing an efficient predictive model for solid slag layer thickness. The model first establishes a foundational BP neural network framework, then introduces the PSO algorithm to perform a global search in the parameter space for optimal initial weights and biases, aiming to minimize network error. Here, weights determine the connection strength between neurons, directly influencing prediction accuracy, while biases regulate the activation range of neurons, enhancing the network’s fitting capability. Compared to traditional BP algorithms, the PSO mechanism effectively mitigates issues like gradient vanishing and local optima. By completing parameter optimization before training, it significantly accelerates convergence and enhances the model’s predictive performance.

**Figure 14:**
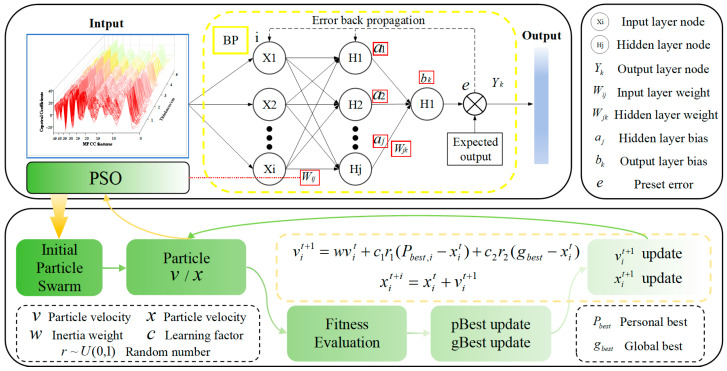
PSO-BPNet Architecture.

To more clearly illustrate the working mechanism of PSO-BPNet, Figure 14 presents a schematic diagram of the overall architecture of the algorithm. The diagram primarily consists of two parts: the upper section shows the topological structure of the BP neural network, illustrating the connection relationships between the input layer, hidden layer, and output layer, as well as the path of error backpropagation. The lower section depicts the iterative process of the PSO algorithm, including particle initialization, velocity and position updates, fitness evaluation, and the update mechanisms for individual best (pBest) and global best (gBest). Through this architecture diagram, the collaborative workflow between the PSO module and the BP network can be clearly observed. The PSO module globally optimizes the network parameters before training, providing the BP network with superior initial values. This effectively mitigates issues such as gradient vanishing and local optima, significantly enhancing the model’s convergence speed and prediction accuracy.

#### 2.3.4. Molten Steel Level Detection Using Eddy Current

Eddy current detection is based on the principle of electromagnetic induction, employing a mutual-inductance eddy current sensor for molten steel level detection. The sensor probe consists of an excitation coil and a detection coil. When low-frequency alternating current is applied to the excitation coil, the generated alternating magnetic field exhibits distinct electromagnetic response characteristics as it penetrates the multi-layered media inside the mold: the upper air layer and solid slag layer (nearly non-conductive) [24] do not generate eddy currents; the intermediate liquid slag layer (electrical conductivity 0.1–0.3 S/cm) [29] produces only weak eddy currents; and the lower molten steel layer (electrical conductivity 750–2500 S/cm) generates significant eddy currents. Since the electrical conductivity of the liquid slag layer is substantially lower than that of molten steel, its influence on the detection results is negligible. Therefore, the level detection primarily manifests as changes in the voltage of the detection coil caused by variations in the relative distance between the molten steel and the sensor.

In practical measurements, the voltage output from the sensor exhibits a nonlinear relationship with the measured distance. The system collects sensor output data through virtual instrumentation, establishes a voltage-distance correspondence lookup table, and employs interpolation to estimate measurement values not included in the table in real time. By comparing the original signal with corrected values to compensate for nonlinear errors, high-precision level detection is achieved.

## 3. Experiments and Results

### 3.1. Hardware Equipment

(1) Millimeter-Wave Radar Sensor:

To reconstruct the topography of the upper surface of the solid slag layer and predict its thickness, a data acquisition system was constructed using the TI IWR1843 millimeter-wave radar module (Texas Instruments, Dallas, TX, USA) and the DCA1000EVM capture card (Texas Instruments, Dallas, TX, USA). The IWR1843 transmits and receives target echo signals, while the DCA1000EVM transmits the raw data in real time to a PC for acquisition and processing.

(2) Eddy Current Sensor:

A self-developed mutual-inductance eddy current sensor (including excitation and detection coils) is used to detect the molten steel level. A signal generator drives the excitation coil to produce the excitation signal, while the detection coil outputs the induced signal carrying molten steel level characteristic information, which is transmitted via a data acquisition card to a PC for processing.

**Figure 15:**
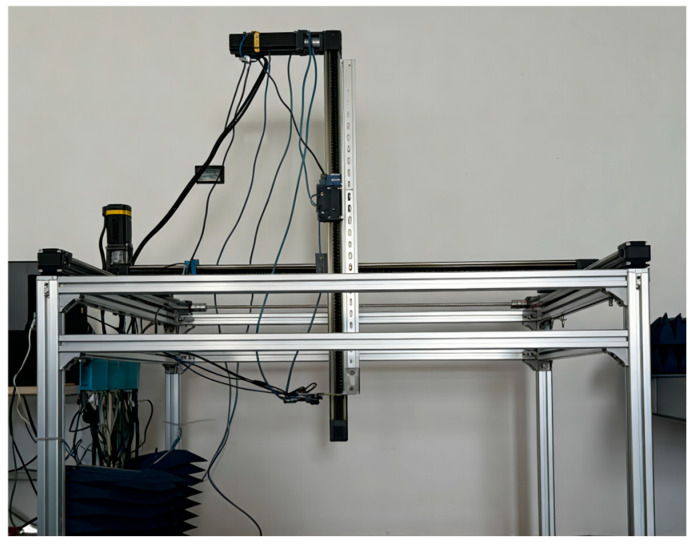
Physical Diagram of the Three-Axis Linear Slide Rail.

(3) Three-Axis Linear Slide Rail:

A high-precision three-axis linear slide rail (integrated with an AMC4030 motion controller; equipment sourced from Chengdu Fuyu Technology Co., Ltd., Chengdu, China) is employed to achieve precise displacement control and data acquisition for the millimeter-wave radar. The millimeter-wave radar module is integrated on the *Z*-axis of the three-axis slide rail. Through an X-Y plane S-shaped path dynamic scanning strategy, spatially differentiated sampling of the slag surface is realized, ensuring scanning coverage and avoiding local repeated measurements. Figure 15 shows the physical diagram of the three-axis linear slide rail.

### 3.2. Reconstruction of the Topography of the Upper Surface of the Solid Slag Layer

**Figure 16:**
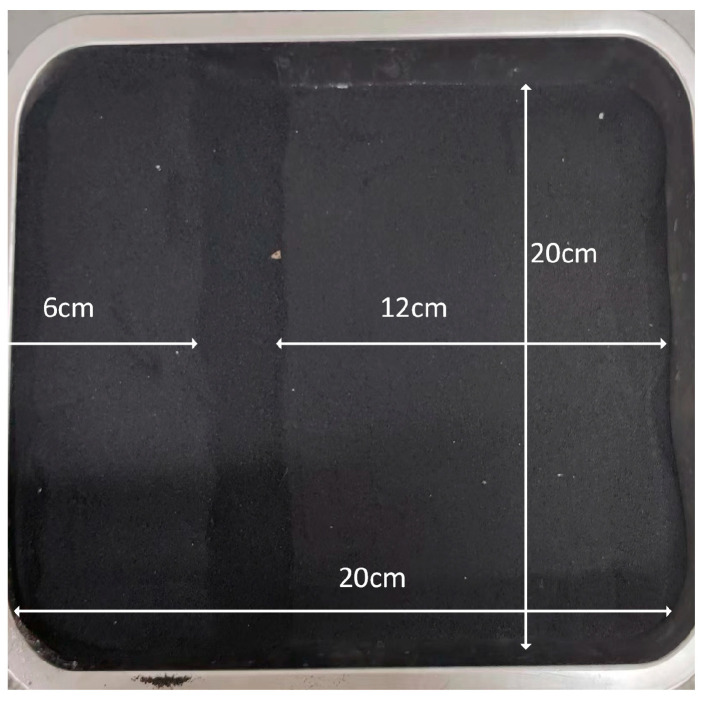
Actual Working Conditions of Mold Slag.

To validate the effectiveness of the millimeter-wave radar in reconstructing the topography of the solid slag layer surface and its thickness measurement accuracy in real industrial scenarios, the following experiment was designed: a steel shell of known geometric dimensions was fully filled with uniform mold slag, and the state of the solid slag layer was meticulously adjusted according to preset three-dimensional topography parameters to simulate actual working conditions (as shown in Figure 16).

**Figure 17:**
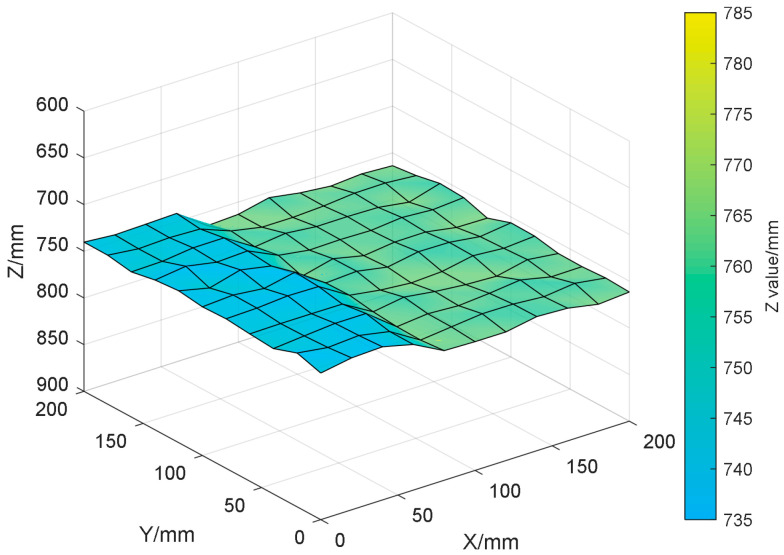
Reconstruction of the Topography of the Upper Surface of the Solid Slag Layer.

The radar module is fixedly installed on the *Z*-axis and scans the upper surface of the solid slag layer in a planned S-shaped trajectory on the X-Y plane for three-dimensional data acquisition. The planned motion trajectory is equally divided at fixed intervals, with each division point serving as a sampling pixel. The sliding platform drives the radar module to collect echo signals point by point, and the FFT-CZT^2^ algorithm is employed for high-precision ranging to obtain accurate distance values from the radar to the upper surface of the solid slag layer at each pixel (measurement error ±1 mm, using the average of three repeated measurements). Based on this distance data, the topography of the upper surface of the solid slag layer is reconstructed (as shown in Figure 17).

### 3.3. Prediction of Solid Slag Layer Thickness

#### 3.3.1. Dataset for Solid Slag Layer Thickness Prediction

To simulate the measurement conditions for mold slag thickness in industrial settings, this study utilized mold slag with real raw material ratios to construct a sample library covering a range of 0 to 5 cm. This sample library comprises a total of 6000 radar data samples, all stored in (.bin) file format.

Within the measurement range from 0 to 5 cm, data points are collected at 1 cm intervals. Each position contains 1000 samples. The dataset was split into training, validation, and test sets in a 7:1:2 ratio. MFCC was used to extract 100-dimensional feature coefficients from each sample, which were then input into the PSO-BP neural network for training.

#### 3.3.2. Training Configuration and Evaluation Metrics

The server configuration used in the laboratory includes an Intel Core i7-14700F CPU (2.10 GHz) and an NVIDIA GeForce RTX 4060 GPU (8 GB). The programming languages employed are Python 3.9 and MATLAB 2023b. The training was conducted for 300 iterations, with inputs being 100-dimensional MFCC feature coefficients. For the solid slag layer thickness prediction task, the detection accuracy and generalization capability of each model were comprehensively evaluated through systematic comparisons of three key metrics: Mean Absolute Error (MAE), Root Mean Square Error (RMSE), and the Coefficient of Determination (R^2^).

#### 3.3.3. Results and Analysis of Solid Slag Layer Thickness Prediction

**Figure 18:**
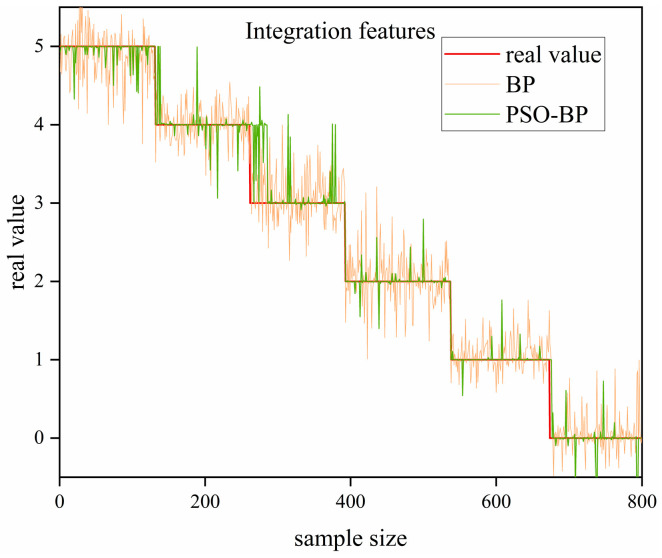
Model Prediction Results.

The prediction of solid slag layer thickness employs the same high-density S-shaped raster scanning trajectory as used in topography reconstruction to acquire raw echo data from the millimeter-wave radar. Mel-Frequency Cepstral Coefficients (MFCC) are applied for feature extraction, effectively characterizing the properties related to solid slag layer thickness, and a corresponding thickness-labeled dataset is constructed. Finally, the extracted MFCC feature vectors are input into the PSO-BPNet prediction model, achieving precise prediction of solid slag layer thickness with the mean absolute error controlled within ±5 mm. As shown in Figure 18, the improved PSO-BPNet model demonstrates significantly superior prediction accuracy compared to the baseline BP model, effectively meeting the training objectives.

**Figure 19:**
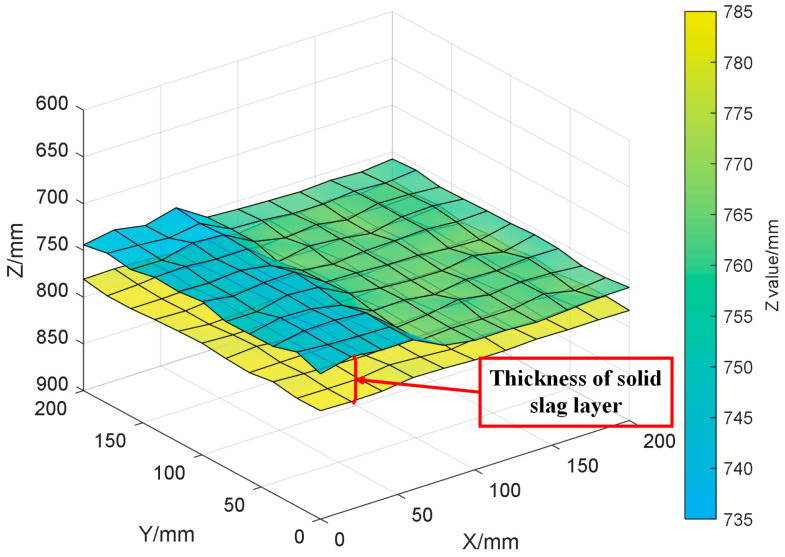
Three-Dimensional Thickness Distribution of the Solid Slag Layer.

Since thickness prediction and topography reconstruction share the same scanning coordinate system and pixel array, after obtaining the three-dimensional topography of the upper surface of the solid slag layer, the predicted thickness value corresponding to each pixel point is spatially fused with its surface height information. This enables the reconstruction of a complete three-dimensional thickness distribution of the solid slag layer, as illustrated in Figure 19.

To validate the performance advantages of the PSO-BPNet algorithm in solid slag layer thickness detection, this study selected four typical network models (1D-CNN, LSTM, ResNet, and VGG19) for comparative experiments, as shown in Table 4.

Compared with other algorithms, the PSO-BPNet algorithm demonstrates significant performance advantages in solid slag layer thickness detection. The algorithm achieved optimal error metrics: the lowest mean absolute error (MAE) of 0.047, a root mean square error (RMSE) of 0.2, and a coefficient of determination (R^2^) of 0.98. This series of metrics fully validates the algorithm’s excellent precision and reliability in predicting thickness variations.

### 3.4. Molten Steel Level Detection

It is challenging to fully replicate industrial mold systems in the laboratory. Therefore, an equivalent simulation platform was constructed: a 20 mm thick copper plate (simulating the copper sidewall of the mold) was used to support the sensor assembly; Q235 steel plates were employed to simulate the molten steel substrate; and tap water (with an electrical conductivity of 0.5–5.0 S/cm, matching the conductive characteristics of the liquid slag layer of 0.1–0.3 S/cm) was selected as the simulation medium for the liquid slag layer.

**Figure 20:**
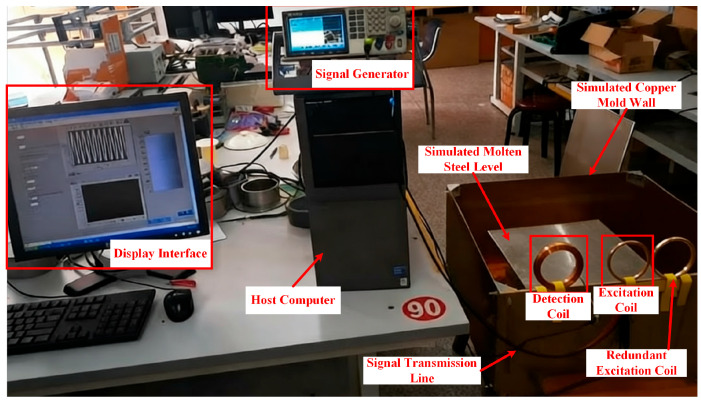
Molten Steel Level Measurement Experiment.

A sinusoidal wave with a frequency of 800 Hz and a peak-to-peak amplitude of 10 V was used as the excitation signal. A host computer control program developed in LabVIEW (LabVIEW 2015) software was employed to drive the data acquisition card, enabling real-time acquisition of the sensor’s response under this excitation. Due to the weak output signal and inherent noise of the sensor, modulation and demodulation techniques were applied for processing: the acquired excitation signal and the sensor response signal were multiplied in real-time within the software, and the product was then passed through a low-pass filter to complete demodulation and filtering, effectively extracting the target information. The processed results were displayed simultaneously in both waveform and numerical forms, achieving high signal-to-noise ratio extraction and intuitive visualization of the signal (as shown in Figure 20).

The experiment employed a dual-coil eddy current sensor (including excitation and detection coils) to perform baseline measurements of the simulated molten steel interface position. Within the full measurement range of 1–120 mm, non-uniformly distributed measurement points were preset: a step size of 2 mm was adopted for the near-distance range of 1–30 mm, while a step size of 3 mm was used for the far-distance range of 30–120 mm, giving a total of 45 measurement points.

**Figure 21:**
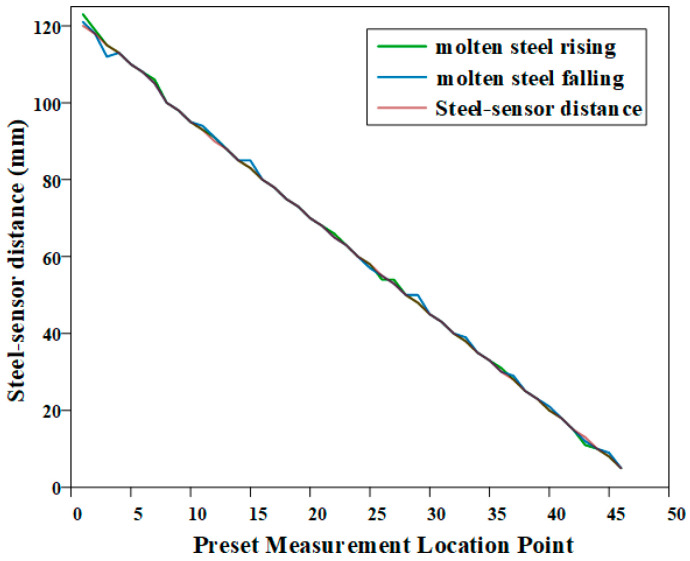
Comparison Curve of Actual Distance vs. Detected Distance for Molten Steel Level.

Based on the results shown in Figure 21, the system maintained an average error of three measurements within ±1 mm during both the rising and falling processes of the molten steel level.

## 4. Conclusions and Future Work

This paper innovatively proposes a method for measuring mold slag thickness in continuous casting molds based on the fusion of millimeter-wave radar and eddy current sensors. Through multi-sensor data fusion, this method achieves precise monitoring of the mold slag condition within the mold: first, the millimeter-wave radar is employed to obtain the three-dimensional topography of the upper surface of the solid slag layer and the thickness distribution of the solid slag layer; simultaneously, the eddy current sensor accurately locates the molten steel interface position; subsequently, via three-dimensional spatial registration, the thickness parameter of the liquid slag layer can be precisely inverted. Additionally, through high-precision reconstruction of the upper surface topography, this approach effectively addresses process control challenges such as uneven material distribution, local material deficiency, and excessive feeding, offering a novel technical solution for quality control in the continuous casting process.

For the three-dimensional topography reconstruction of the upper surface of the solid slag layer, the FFT-CZT^2^ high-precision ranging algorithm is proposed. This algorithm adopts a three-level progressive processing architecture, achieving a measurement accuracy of ±1 mm. In terms of solid slag layer thickness prediction, MFCC radar intermediate-frequency signal feature extraction and an improved PSO-BPNet algorithm are innovatively integrated to establish a prediction model for the thickness of the mold slag solid layer. Experimental validation confirms its maximum prediction error to be within ±5 mm. Additionally, a molten steel level detection system based on eddy current sensors was developed, with experimental measurement results consistently stable within ±1 mm. Through multi-parameter data fusion, the thickness parameter of the liquid slag layer can be precisely inverted.

This study validates the effectiveness of millimeter-wave radar and eddy current sensors in detecting key parameters within continuous casting molds. The results demonstrate that millimeter-wave radar exhibits excellent detection performance for non-dielectric materials in complex environments. Compared to traditional contact-based detection methods, millimeter-wave radar enables non-destructive and non-contact detection. The research confirms that the multi-sensor fusion technology based on eddy current sensors and millimeter-wave radar significantly enhances detection accuracy and system stability, demonstrating stronger environmental adaptability. Future research will focus on the integrated optimization of the sensor system, high-temperature environment experiments, and further validation on actual industrial production lines. With continuous improvements in the technical solution, this method is expected to provide real-time and precise full cross-section monitoring for continuous casting processes, offering significant engineering and practical value for advancing the development of smart steel plants.

## Figures and Tables

**Table 1:** Initial Parameter Settings for the Eddy Current Detection Simulation Model.

Name	Parameter	Name	Parameter
Excitation Coil Radius (mm)	60	Detection Coil Radius (mm)	60
Excitation Coil Inner Diameter (mm)	45	Detection Coil Inner Diameter (mm)	45
Number of Turns in Excitation Coil (N)	400	Number of Turns in Detection Coil (N)	4500
Coil Material	Copper	Coil Wire Cross-Sectional Area (m^2^)	1 × 10^−6^
Molten Steel Measurement Range (mm)	50–60	Solid Slag Thickness (mm)	0–30
Liquid Slag Thickness (mm)	0–20	Copper Wall Thickness (mm)	8
Liquid Slag Relative Electrical Conductivity (S/m)	100	Solid Slag Relative Electrical Conductivity (S/m)	1
Molten Steel Relative Electrical Conductivity (S/m)	710,000	Copper Wall Relative Electrical Conductivity (S/m)	5.998 × 10^7^
Air Relative Electrical Conductivity (S/m)	1	Coil Geometry	Circle

**Table 2:** Relationship between Induced Voltage Variation and Excitation Frequency.

Excitation Frequency/Hz	Induced Voltage Variation/mV
500	41.4
800	41.2
1000	40.6
1500	40.4
2000	40.3
2500	40.2
3000	40.3
10,000	39.6

**Table 3:** Mel Filter Bank Parameters.

Name	Parameters
Filter Number	100
Frequency Range	0–7000 Hz
Center Frequency Distribution	Uniformly distributed on the Mel scale, non-uniformly distributed on the Hertz scale
Sampling Rate	14,000 Hz
Filter Shape	Triangular bandpass
Overlap Rate	50%

**Table 4:** Model Comparison.

Model	MAE	RMSE	R^2^
1D_CNN	0.32	0.46	0.93
LSTM	0.23	0.33	0.96
ResNet	0.22	0.3	0.97
VGG19	0.31	0.44	0.93
BP	0.2	0.29	0.97
**POS-BPNet**	**0.047**	**0.2**	**0.98**

## Data Availability

The datasets used and analyzed in the current study will be available after the paper is accepted.

## Abbreviations

The following abbreviations are used in this manuscript:

MFCC	Mel-Frequency Cepstral Coefficients
RCS	Radar Cross-Section
MAE	Mean Absolute Error
RMSE	Root Mean Square Error
